# Optimal predictive neuro-navigator design for mobile robot navigation with moving obstacles

**DOI:** 10.3389/frobt.2023.1226028

**Published:** 2023-08-09

**Authors:** Mahsa Mohaghegh, Samaneh-Alsadat Saeedinia, Zahra Roozbehi

**Affiliations:** ^1^ School of Engineering, Computing and Mathematical Sciences, Auckland University of Technology (AUT), Auckland, New Zealand; ^2^ Faculty of Design and Creative Technologies, AUT, Auckland, New Zealand; ^3^ School of Electrical Engineering, University of Science and Technology (IUST), Tehran, Iran

**Keywords:** navigation, optimization, MPC, stability, neural network, dynamic environment

## Abstract

**Introduction:** The challenge of navigating a Mobile robot in dynamic environments has grasped significant attention in recent years. Despite the available techniques, there is still a need for efficient and reliable approaches that can address the challenges of real-time near optimal navigation and collision avoidance.

**Methods:** This paper proposes a novel Log-concave Model Predictive Controller (MPC) algorithm that addresses these challenges by utilizing a unique formulation of cost functions and dynamic constraints, as well as a convergence criterion based on Lyapunov stability theory. The proposed approach is mapped onto a novel recurrent neural network (RNN) structure and compared with the CVXOPT optimization tool. The key contribution of this study is the combination of neural networks with model predictive controller to solve optimal control problems locally near the robot, which offers several advantages, including computational efficiency and the ability to handle nonlinear and complex systems.

**Results:** The major findings of this study include the successful implementation and evaluation of the proposed algorithm, which outperforms other methods such as RRT, A-Star, and LQ-MPC in terms of reliability and speed. This approach has the potential to facilitate real-time navigation of mobile robots in dynamic environments and ensure a feasible solution for the proposed constrained-optimization problem.

## 1 Introduction

Neural networks and Model Predictive Controller (MPC) are two powerful techniques in robotics, especially in the path planning of mobile robots. Neural networks are computational algorithms, mimicking human brain functions (Egmont-Petersen et al., 2002), while MPC is a control technique that predicts the future behavior of a system based on a mathematical model. Both approaches are popular methods for planning the path of a mobile robot with the aim of optimizing the path and avoiding obstacles. However, both of them have different strengths and weaknesses. One of the highlighted capabilities of neural networks, in the field of path planning, is their ability to determine the optimal path by training on a set of data from previous successful paths and generalizing its pattern to a new environment based on its input, such as sensory data from the robot’s sensors. On the other hand, MPC uses a mathematical model of the robot’s dynamics and constraints, such as speed limits and obstacle avoidance, to predict the optimal path. This matter improves the performance of MPCs in comparison to neural networks in more complex dynamics and constraints. Although, achieving this requires modeling and accurate calculations (Yao et al., 2015).

Depending on the specific requirements, engineers may find both neural networks and MPC to be highly useful. In fact, a combination of these methods can provide robotics experts with even greater capabilities to enhance the efficacy and reliability of path planning (Karur et al., 2021; Askari et al., 2022).

In the realm of path planning for mobile robots, it is imperative to not overlook the significance of model-free techniques, even while capitalizing on model-based approaches such as MPCs. Although model-free control methods, exemplified by Q-learning and its derivatives, have been extensively studied in mobile robot navigation (Du et al., 2022), their computational intensity may render them unsuitable for systems with stringent constraints or safety prerequisites. In contrast, MPC provides a systematic framework for addressing constraints and objectives, making it a versatile tool for navigating mobile robots in intricate and dynamic environments (Xiao, 2022). Moreover, MPC can be integrated with learning-based techniques, such as neural networks, to enhance prediction model accuracy and attain high-performance path tracking (Stano, 2022). Additionally, incorporating exploration-exploitation-based adaptive principles into policy-based and value-based methods constitutes an additional strategy for model-free management (Song and Scaramuzza, 2020). Nonetheless, these techniques can be exceedingly computational and may necessitate vast amounts of data for training.

The literature review reveals that most of the methods in recent research have utilized neural networks to predict the environment for robot navigation (Bency et al., 2019; Haider et al., 2022; Lee and Yusuf, 2022; Kim et al., 2018; Wang et al., 2017; Quan et al., 2020). However, in this study, we propose a novel approach that combines neural networks with model predictive controller to solve optimal control problems locally near the robotic system. Specifically, we utilize neural networks as a function approximator to estimate the system dynamics and cost function, which are then used by the model predictive controller algorithm to solve the optimal control problem.

In a recent study a learning-based nonlinear model predictive controller (LB-NMPC) algorithm is presented to improve vision-based mobile robot path-tracking in challenging outdoor environments (Limon et al., 2017). This approach combines learning-based techniques with MPC to enhance the accuracy of the prediction model and achieve high-performance path tracking. Saeedinia and Tale Masouleh (2022) proposed a new navigation control scheme for a three-wheel omnidirectional robot that takes into account the dynamic constraints to achieve stable and efficient navigation, combining multi-modal MPC and Q-learning to improve the accuracy of the prediction model and handle system constraints in the control process. Additionally, there have been recent breakthroughs in deep reinforcement learning-based MPC controllers for mobile robots that have the capability of moving omnidirectionally. These controllers utilize neural networks to estimate the robot’s future state and generate optimal control inputs using MPC. Experimental validation on real robots has demonstrated improved performance compared to traditional MPC controllers (Lee and Yusuf, 2022; Ramezani et al., 2023). As another work, we can refer to a real-time neural MPC has been proposed for quadrotors and agile robotic platforms, which makes use of neural networks as dynamic model to optimize control inputs in real-time (Salzmann et al., 2023).

While most of the existing literature focuses on using neural networks to predict the environment, our work emphasizes utilizing neural networks to solve optimal solutions locally near the robot. This approach confers advantages such as increased accuracy and efficacy in the navigation of robotic systems.

Other recent research has also been conducted regarding path planning and navigation for mobile robots. For example, a study proposed an improved deep reinforcement learning algorithm for path planning in large-scale dynamic environments, considering the avoidance of dynamic obstacles (Xie et al., 2021). Another study explored the use of object detection using CNNs for model-predictive control of omnidirectional mobile robots in logistic environments (Achirei et al., 2023). These studies highlight the ongoing research efforts in developing advanced techniques for mobile robot navigation.

On the other hand, the process of identifying the most efficient trajectory for a mobile robot is inherently characterized by non-linearity and occasionally non-convexity, thereby restricting the applicability of standard convex optimization tools in the context of MPCs applications. In this regard, a MPC that is based on neural networks (Neuro-MPC) can prove to be particularly advantageous when navigating complex environments in which dynamic obstacles cannot be readily modeled using convex optimization tools (Deits and Tedrake, 2014).

Although the combined method mentioned above offers numerous advantages, it is crucial not to overlook the importance of system stability. This issue cannot be neglected and must be taken into consideration. The stability of Neuro_MPC is a critical issue in path planning because instability can lead to unexpected behavior of the autonomous system, which may cause accidents or damage to the environment (Yang et al., 2010; Cheng et al., 2019). Several methods have been proposed to guarantee the stability of Neuro_MPC, such as Lyapunov-based stability analysis and robust control techniques. These methods ensure that the MPC controller generates control actions that steer the system towards a safe and stable trajectory. Thus, ensuring the stability of Neuro_MPC is essential to prevent unexpected behavior and ensure the safety of both the autonomous system and its environment.

In order to tackle the challenges at hand, we have developed a novel heuristic log-concave MPC navigation problem that takes into account dynamic and kinematic constraints, and guarantees convergence based on Lyapunov stability theory. To solve this problem, we have designed a new structure for a recurrent neural network that is inspired by the NN constrained formulation presented in (Villarrubia et al., 2018), and utilizes a screen searching based approach.

Our exploration revolves around a three-wheeled omnidirectional robot that uses a differential drive scheme to control its motion. This distinctive robot architecture confers greater dexterity and accuracy in traversing intricate environments, particularly when confronted with mobile obstacles. Our work employs the built-in tracking controller, which is based on the controller devised by Peñaloza-Mejía et al. (2015). This controller is a PID controller, effectively steers the robot’s locomotion while accommodating velocity constraints. Nevertheless, in order to ensure compatibility with the environment and surmount limitations associated with mobile obstacles, we have implemented MPC to govern the entire system, in conjunction with the integrated tracking controller. In our research, we have employed MPC to optimize the robot’s movement and positioning in real-time, taking into account the existence of mobile obstacles within the surroundings. We have furnished further explication on the functionality of the MPC controller in the following sections.

This paper is organized into several sections to provide a comprehensive overview of our approach. In Section 2, we introduce the Materials and Methods, which includes a detailed description of the navigation strategy, a new definition of the log-concave navigation MPC problem, and proposing a novel Recurrent Neural Network structure to solve the complex nonlinear problem. In Section 3, we compare the results of our proposed navigation strategy with those of well-known classical and reactive methods, namely, RRT, A-star and LQ-MPC methods. Finally, in Section 4, we discuss the simulation results and draw conclusions.

## 2 Materials and methods

**FIGURE 1:**
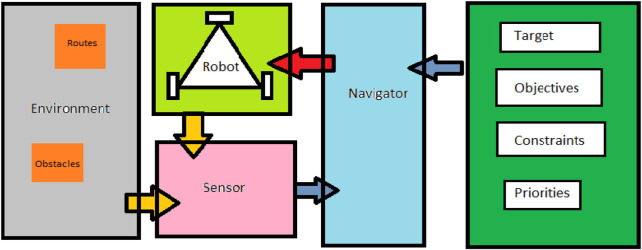
A general navigation system interactions.

The general navigation system for robots involves various factors such as the positions of the target and the robot, the environment’s characteristics and arrangements, and the constraints and objectives. (See Figure 1 for a visual representation of these interactions.).

When it comes to global path planning, having knowledge of the environment and its characteristics is crucial in developing optimal strategies. This is why the environment is monitored globally in optimal approaches to reduce distance costs. However, local information is sufficient in local navigation methods to reduce the cost of time response for real-time implementation. Unfortunately, this may not always consider distance costs appropriately, which is why a trade-off between local and global advantages is necessary.

**FIGURE 2:**
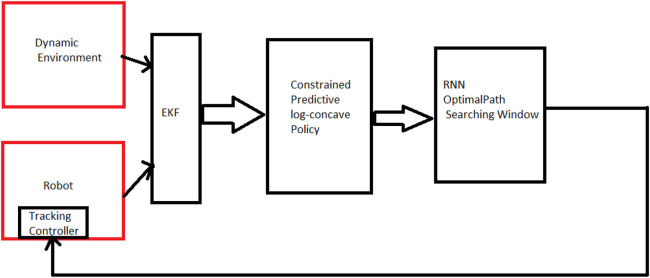
Internal structure of the proposed path planning system.

To address this issue, researchers have developed heuristic algorithms that benefit both strategies. Our paper proposes a combination of local predictive path planning methods with global information about the environment. (See Figure 2 for the internal structure of our proposed path planning system.).

The term “local information” refers to data collected around the robot’s current position, while “global environment data” refers to information about the robot’s overall environment. For predicting the behavior of moving obstacles, both local and global information are used. The local data predicts immediate behavior, while global environment data predicts future behavior. This tactic reduces risk, minimizes computational expenses, and presents a complete comprehension of the system.

Our approach involves processing the received global image to label obstacle positions as 1 and the rest as 0. We then estimate the locations of dynamic obstacles in future time intervals to avoid collisions using multiple EKF estimators. The constrained predictive log-concave policy block is the center of rewarding the policy, designed to maximize the award when the path is optimal and minimize it in the presence of a high risk of collision.

We also designed an Recurrent Neural Network (RNN) block to receive updated information on the position classified data and evaluate the next state policy cost of the robot’s neighborhood, considering a conservative collision avoidance constraint compatible with finite state prediction steps. By combining local and global information, our proposed path planning system offers a more comprehensive and effective approach to robot navigation.

In order to assess the effectiveness of our navigation strategy, we utilized Python 3.10 to simulate three randomly moving obstacles, as well as a real three-wheeled omnidirectional mobile robot (OMR) dynamic model with a built-in tracking controller introduced by (Peñaloza-Mejía et al., 2015). Our goal was to create a realistic indoor environment and robot dynamic behavior simulation, allowing us to thoroughly investigate the proposed navigator performance. To achieve this, we utilized the robot dynamic model and the Kalman Filter updating equations (Blanchard, 2007) as the real robot dynamics indicator and the observer component of the navigation system, respectively.

By and large, we used the EKF algorithm for obstacle tracking and estimation. The EKF algorithm is used to estimate the position and velocity of obstacles and the robot in the environment, which are utilized as inputs to the MPC algorithm to generate control actions for the robot. The EKF algorithm considered the position and velocity of the obstacles and predicted their next position by predicting one step ahead.

The MPC algorithm was responsible for generating control actions that steer the robot towards the target while avoiding collisions with obstacles. The MPC algorithm used the estimated positions and velocities of obstacles obtained from the EKF algorithm as inputs to generate control actions for the robot. The cost function used in the MPC algorithm was designed to balance the tradeoff between achieving the desired goal and avoiding obstacles while minimizing energy consumption.

The RNN structure is designed as a local optimization solver for the nonlinear MPC problem. Specifically, the RNN is used to estimate the system dynamics and cost function, which are then used as inputs to the MPC algorithm to solve the optimal control problem. The RNN is trained to minimize the cost function in the Lagrangian form by adjusting the input control to the system at each time step, such that the constraints are satisfied and the state of the system approaches the desired reference trajectory.

### 2.1 Environment simulation and data collection

This section provides a comprehensive description of the preprocessing and analysis of the sensor data utilized in our simulation study. In consideration of the EKF converging process and RNN learning and training time samples, we utilized 70 sampling epochs as the training dataset, using data recursively, and the remaining epochs for testing.

The sensor data is simulated as flag data, which detects obstacles from a distance of 0.4 m. If obstacles are located in front of the sensor, the flag is set to 1, otherwise, it is set to 0.

To generate the input data for the learning algorithm, we used a camera mounted on the ceiling of the room to detect the position and color of obstacles in the environment. The position of the robot and obstacles were labeled as global positions, and the obstacles were represented as binary values, with 1 indicating the presence of an obstacle and 0 indicating no obstacle at that position. We also added normal random noise to the positions of the robot and obstacles to simulate the measuring noise in the sensor data.

To analyze the sensor data, we first pre-processed the data to remove noise and outliers and normalized the data to ensure that the learning algorithm could effectively learn from the data. We also used data augmentation techniques to increase the size of the dataset and improve the generalization ability of the learning algorithm.

Next, we used the Extended Kalman Filter (EKF) algorithm to estimate the position and velocity of obstacles in the environment and the robot. The EKF algorithm considered the position and velocity of the obstacles and estimated their next position by predicting one step ahead. Obstacles were designed with random velocity and shape to simulate a realistic environment. If any new obstacles were detected, a new EKF was started to estimate its position. If the predicted location and sensor data were not compatible, the sensor data was given priority, and the robot changed its direction by 45° in each step while the sensor flag was set to zero to show the lack of obstacles nearby. Finally, we used the pre-processed sensor data as input to the learning algorithm and trained the algorithm to predict the position and velocity of obstacles in the environment.

### 2.2 Mobile robot model

To simulate realistic dynamic behavior of a mobile robot, a real three-wheeled OMR Dynamic model parameters and internal tracking control law, given by (Peñaloza-Mejía et al., 2015) are utilized. The dynamic model of OMR is described as follows:

$$M\left(q^{∙∙}\right)q+C\left(q^{∙},q^{∙}\right)q=B\left(q\right)\tau$$ (1)

The inertial matrix, the Coriolis and centrifugal force vector, and the input matrix, defined as follows:

$$\;M\left(q\right)=\left[\begin{array}{ccc} m+\frac{3J}{2r^{2}} & 0 & 0\\ 0 & m+\frac{3J}{2r^{2}} & 0\\ 0 & 0 & I_{z}+\frac{3JL^{2}}{r^{2}} \end{array}\right]$$

$$C\left(q^{∙},q\right)=\left[\begin{array}{ccc} 0 & {\frac{3J}{2r^{2}}}^{∙}\varphi_{w} & 0\\ -{\frac{3J}{2r^{2}}}^{∙}\varphi_{w} & 0 & 0\\ 0 & 0 & 0 \end{array}\right]$$ (2)

$$B\left(q\right)=\left[\begin{array}{ccc} -\frac{1}{2r}\left(\sin\left(\varphi_{w}\right)-\sqrt{3}\cos\left(\varphi_{w}\right)\right) & \frac{1}{r}\sin\left(\varphi_{w}\right) & -\frac{1}{2r}\left(\sin\left(\varphi_{w}\right)+\sqrt{3}\cos\left(\varphi_{w}\right)\right)\\ \frac{1}{2r}\left(\sin\left(\varphi_{w}\right)+\sqrt{3}\cos\left(\varphi_{w}\right)\right) & -\frac{1}{r}\cos\left(\varphi_{w}\right) & \frac{1}{2r}\left(\sin\left(\varphi_{w}\right)-\sqrt{3}\cos\left(\varphi_{w}\right)\right)\\ \frac{L}{r} & \frac{L}{r} & \frac{L}{r} \end{array}\right]$$

The parameters of the model are stated in Table 1, and the tracking control law is described in (Peñaloza-Mejía et al., 2015).

**TABLE 1:** The Real Robot Model Parameters, taken by (Peñaloza-Mejía et al., 2015).

Parameters	Units	Quantities
m	kg	11.83
r	m	0.0625
L	m	0.287
I_z_	kg·m^2^	0.0127
J	kg·m^2^	5.82E-4

### 2.3 Prediction of the future states

This paper employs m+1 Kalman filters concurrently to forecast the future states of m obstacles, as well as the robot’s position and velocity, based on the mean square error of the estimation. The Kalman Filter Parameter estimation laws are derived from (Blanchard, 2007), and the regressor for the obstacles is defined as follows:

$$X_{oi}\left(k\right)=\left[x_{oi\left(k-1\right)}\;v_{oi\left(k-1\right)}\right]$$ (3)

The estimated future states of the robot are utilized to predict the future screen of the environment. Moreover, to predict future states of the robot, a Kalman filter is implemented to estimate the parameters of an Auto-Regressive Exogenous Model, which is considered as the dynamic constraint of the robot in the proposed MPC navigator.

### 2.4 The log-convex navigation MPC definition

This section addresses a common challenge in obstacle avoidance, which involves dynamic and kinematic constraints within a log-convex MPC framework. In order to tackle this issue, we have identified several key rules that are typically applied in navigation problems. These rules have been translated into mathematical expressions that can be incorporated into the MPC cost function and constraints.

Rule one emphasizes the importance of taking the shortest route possible, while rule two highlights the value of maintaining a safe distance from obstacles. Rule three prioritizes the use of sensor information over image processing for obstacle detection, and rule four stresses the need for accurate tracing at all times. Rule five reminds us to adhere to the maximum allowable speed, and rule six emphasizes the importance of ensuring system stability.

To incorporate these rules into our MPC framework, we have developed a set of linear constraints and kernel functions that allow us to evaluate their relative importance on a scale of zero to one. Rules one to five are incorporated into a constrained MPC structure, which is defined as follows:

$$\min_{\varepsilon ,\vartheta }J_{\infty }=\sum_{n=k}^{\infty }\underset{J_{n}}{\underbrace{\int_{n\Delta t}^{\left(n+1\right)\Delta t}J\left(t\right)dt}}=\sum_{n=k}^{k+N}J_{n}+\sum_{n=k+N+1}^{\infty }J_{n}=\sum_{n=k}^{k+N}J_{n}+I\left(\left(N+1\right)\Delta t\right)$$ (4-1)

$$\begin{array}{l} S.t.\\ {\hat{Y}}_{k+1}={\hat{\gamma }}^{T}\left[\begin{array}{c} Y_{k}\\ U_{k} \end{array}\right],\\ \left|P_{k}-O_{ik}\right|>2\left(2r\right)\\ \left\{\begin{array}{l} O_{ik}=\left[\begin{array}{cc} I_{3\times 3} & 0_{3\times 3} \end{array}\right]{\left[\begin{array}{ccc} X_{oi}\left(k+1\right) & \cdots & X_{oi}\left(k+N\right) \end{array}\right]}_{6\times N}\\ Y_{p}={\left[\begin{array}{ccc} {\hat{Y}}_{k+1} & \cdots & {\hat{Y}}_{k+N} \end{array}\right]}_{6\times N}\\ P_{k}=\left[\begin{array}{cc} I_{3\times 3} & 0_{3\times 3} \end{array}\right]Y_{p} \end{array}\right.\\ \left|{\hat{\varphi }}_{w}\left(k\right)\right|\geq Flag\times \left(\frac{\pi }{4}\right)\\ \begin{array}{cc} \left|\left[\begin{array}{cc} {\vec{0}}_{3\times 3} & {\vec{1}}_{3\times 3} \end{array}\right]Y_{k_{6\times 1}}\right|\leq & \left[\begin{array}{c} V_{\max}.\cos\left(\varphi_{w}\left(k\right)\right)\\ V_{\max}.\sin\left(\varphi_{w}\left(k\right)\right)\\ \omega_{\max} \end{array}\right] \end{array} \end{array}$$ (4-2)

where $J\left(t\right)$ is defined as follows:

$$\begin{array}{ll} J\left(t\right) & =1-\exp\left(-0.5{\left(Y_{k\Delta t}-Y_{f}\right)}^{T}\Lambda^{-1}\left(Y_{k\Delta t}-Y_{f}\right)\right)\\ & \;+\exp\left(\left(-0.5{\left(Y_{k\Delta t}-X_{oi}\right)}^{T}\Lambda^{-1}\left(Y_{k\Delta t}-X_{oi}\right)\right)\right)\\ & \;+1-\exp\left(-0.5\left({U_{k\Delta t}}^{T}\right)\Lambda^{-1}\left(U_{k\Delta t}\right)\right) \end{array}$$ (5)

where $Y\left(t\right)=Y\left(k\Delta t\right)=Y_{k}$ is discrete-time sampling. In which $\Lambda^{-1}$ is defined based on the constraint of the robot dynamic and environment limitations.

$$\Lambda^{-1}=\left[\begin{array}{cc} \left[\begin{array}{ccc} \frac{1}{d_{x}} & 0 & 0\\ 0 & \frac{1}{d_{y}} & 0\\ 0 & 0 & \frac{1}{d_{\theta }} \end{array}\right] & 0_{3\times 3}\\ 0_{3\times 3} & \left[\begin{array}{ccc} \frac{1}{V_{\max}} & 0 & 0\\ 0 & \frac{1}{V_{\max}} & 0\\ 0 & 0 & \frac{1}{\omega_{\max}} \end{array}\right] \end{array}\right]$$ (6)

Where *d* indicates maximum possible movement in the Environment toward the (x-y) axis. In addition, the maximum possible rotation angle is shown by $d_{\theta }$ . *V*_*max*_ denotes the maximum possible velocity for the robot, according to the environment and robot dynamic. $\omega_{\max}$ is the maximum possible angular velocity. $Y_{k}={\left[\begin{array}{cc} q & \dot{q} \end{array}\right]}^{T}$ indicates position and velocity vector of the robot dynamic at time step k. *U*_*k*_ is the control law, i.e., the generated path. ${\hat{\gamma }}^{T}$ is the estimated model parameter by KF. ${\hat{\varphi }}_{w}\left(k\right)$ is the estimated robot angle and ${\hat{Y}}_{k+1}$ is the one-step-ahead prediction of $Y_{k}$ . *Flag* is 1 if the built-in sensors detect obstacles around the robot, otherwise, it is zero, to deal with uncertainties and possible faults of environment predictions. This item is considered to avoid the robot collision with obstacles, in the KF failure situations.

The cost function used in this study is a key component of the model predictive controller (MPC) algorithm that controls the navigation of the robot. It is designed to balance the tradeoff between achieving the desired goal (i.e., reaching the target point) and avoiding obstacles while minimizing the energy consumption of the robot. The position cost function is given by the term $1-\exp\left(-0.5{\left(Y_{k\Delta t}-Y_{f}\right)}^{T}\Lambda^{-1}\left(Y_{k\Delta t}-Y_{f}\right)\right)$ . This term penalizes the distance between the robot’s current position $Y_{k\Delta t}$ and the target point $Y_{f}$ . The collision cost function is given by $\exp\left(\left(-0.5{\left(Y_{k\Delta t}-X_{oi}\right)}^{T}\Lambda^{-1}\left(Y_{k\Delta t}-X_{oi}\right)\right)\right)$ , and the energy cost function is given by $1-\exp\left(-0.5\left({U_{k\Delta t}}^{T}\right)\Lambda^{-1}\left(U_{k\Delta t}\right)\right)$ , here is the command requests to the robot to change its position and orientations. Constraints of the navigation problem, given in Eq. 4-2 are break down to system dynamic constraint, local and global collision avoidance, using sensor data, and kinematic constraint.

This paper utilizes ARMAX model to estimate velocity and position of the robot as follows:

$$\begin{array}{l} Y_{k+1}=aY_{k}+bU_{k}+e_{k}\begin{array}{cc} , & e_{k}=N\left(0,1\right) \end{array}\\ E\left\{Y_{k+1}\right\}={\underset{\gamma }{\left[\begin{array}{c} a\\ b \end{array}\right]}}^{T}\underset{\varphi }{\left[\begin{array}{c} Y_{k}\\ U_{k} \end{array}\right]}=\gamma \varphi \end{array}$$ (7)

Where Y_k_ denotes the position and velocity of the robot at kth sample time, and $e_{k}$ is a disturbance with normal distribution. φ is the input regressor, and the next states of the robot are calculated as below:

$$C1:{\hat{Y}}_{k+1}=\gamma {\left(:,1\right)}^{T}Y_{k}+\gamma {\left(:,2\right)}^{T}U_{k}$$ (8)

Considering constant control input in each sample time, we define N-horizon prediction of states vector as follows:

$$\begin{array}{l} Y_{p}={\left[\begin{array}{ccc} Y_{k+1} & \cdots & Y_{k+N} \end{array}\right]}_{6\times N}\\ P_{k}=\left[\begin{array}{cc} I_{3\times 3} & 0_{3\times 3} \end{array}\right]Y_{p} \end{array}$$ (9)

Where *P*_*k*_ is the prediction of N next positions of the robot.

Suppose that *D*_*oi*_ *= [x*_*oi*_ *, v*_*oid*_ *]* ^*T*^ denotes to the i_th_ obstacle’s position and velocity vector in the environment at the global coordination. Obstacles’ states predications are calculated below:

$$\begin{array}{l} X_{oi}\left(k+1\right)=\left[\begin{array}{cc} I_{3\times 3} & \Delta t\times I_{3\times 3}\\ 0_{3\times 3} & I_{3\times 3} \end{array}\right]X_{oi}\left(k\right)\\ O_{ik}=\left[\begin{array}{cc} I_{3\times 3} & 0_{3\times 3} \end{array}\right]{\left[\begin{array}{ccc} X_{oi}\left(k+1\right) & \cdots & X_{oi}\left(k+N\right) \end{array}\right]}_{6\times N} \end{array}$$ (10)

To show the constraint of the robot and obstacles collision avoidance, we define the below criteria:

$$C2:\left|P_{k}-O_{ik}\right|>\Omega ,\;\Omega >0$$ (11)

Where Ω is the conservative distance parameter, it should be determined based on the maximum estimated velocity of the robot, and obstacles to handling prediction’s uncertainties. Where Ω is the conservative distance parameter, it should be determined based on the maximum estimated velocity of the robot, and obstacles to handling prediction’s uncertainties, here is considered $2\left(2r\right)$ .

Due to possible prediction faults and uncertainties, the importance of checking robot obstacle detection sensors increases, so we consider a Flag to detect an obstacle. Flag = 1 indicates an obstacle is nearby the robot, and Flag = 0 reveals the lack of obstacle around the robot.

$$C3:\left|Y\left(3\right)\right|\geq Flag\times \left(\frac{\pi }{4}\right)$$ (12)

This inequality can also be considered as a fitness neighborhood searching function, instead of intending it as another constraint of the MPC problem. This substitution helps to preserve convexity of the problem, when convex optimization solver is utilized.

Kinematic constraints should be regarded in the navigation strategy. This study considers a mere velocity constraint.

$$C5:\begin{array}{cc} \left|\left[\begin{array}{cc} 0 & 1 \end{array}\right]Y\right|\leq & \left[\begin{array}{c} V_{\max}.\cos\left(\theta \right)\\ V_{\max}.\sin\left(\theta \right)\\ \omega_{\max} \end{array}\right] \end{array}$$ (13)

### 2.5 Designing control law with stability guarantee

This section provides an error feedback control law, $U\left(t\right)=F\left(Y\left(t\right)-Y_{f}\right)$ which is able to ensure the stability of MPC problem, (4). To this aim, consider the candidate Lyapunov function as follows:

$$J_{\infty }=\int_{t}^{\infty }\left(\begin{array}{c} 1-\exp\left({-0.5\left(Y\left(t\right)-Y_{f}\right)}^{T}\Lambda^{-1}\left(Y\left(t\right)-Y_{f}\right)\right)\\ +\exp\left(\left({-0.5\left(Y\left(t\right)-X_{oi}\right)}^{T}\Lambda^{-1}\left(Y\left(t\right)-X_{oi}\right)\right)\right)\\ +1-\exp\left({-0.5\left(Y\left(t\right)-Y_{f}\right)}^{T}F^{T}\Lambda^{-1}F\left(Y\left(t\right)-Y_{f}\right)\right) \end{array}\right)dt$$ (14)

F is discrete-time error-feedback gain In order to ensure stability in a navigation system, it is necessary to determine the criterion for stability guarantee. This can be achieved by calculating the derivative of the defined Lyapunov function, which should be negative. By simplifying the Lyapunov inequality constraint of stability, it is possible to determine whether the navigation system is stable.

Stability in navigation is crucial for ensuring that a robot can reach its intended destination without any collisions. To simplify the stability criterion, a linear approximation of the cost function is used, taking into account the limited arguments of the kernel functions, which are restricted to values of 0 and 1. By following these steps, it is possible to ensure that the navigation system is stable and reliable, allowing for safe and efficient navigation.

$$\begin{array}{ll} \frac{\partial J_{\infty }\left(t\right)}{\partial t} & =H+H^{T}\\ H & =0.5{\left(\dot{Y}\right)}^{T}\left(-\Lambda^{-1}\left(X_{oi}-Y_{f}\right)+F^{T}\Lambda^{-1}F\left(Y-Y_{f}\right)\right)\\ \frac{\partial J_{\infty }\left(t\right)}{\partial t} & \leq \int_{t}^{\infty }\left(H\left(t\right)+H^{T}\left(t\right)\right)\partial t\\ & <\underset{I\left(t\right)}{\underbrace{1-\left(-Y^{T}\Lambda^{-1}\left(X_{oi}-Y_{f}\right)+\left(Y^{T}\right)F^{T}\Lambda^{-1}F\left(Y-Y_{f}\right)\right)}}<0 \end{array}$$ (15)

$$\begin{array}{l} \;I\left(t\right)=1-{\left[\begin{array}{c} -Y\left(t\right)\\ Y\left(t\right) \end{array}\right]}^{T}\left[\begin{array}{cc} \Lambda^{-1} & 0\\ 0 & F^{T}\Lambda^{-1}F \end{array}\right]\left[\begin{array}{c} X_{oi}-Y_{f}\\ Y-Y_{f} \end{array}\right]<0,\\ \Lambda^{-1}>0,\;F^{T}\Lambda^{-1}F>0 \end{array}$$

### 2.6 Recurrent neural network design to seek the near optimal path

This section is assigned to map the newly defined optimization problem into the searching-based recurrent Neural Network structure. To this aim, consider a general Lagrangian constrained optimization problem as follows (Ramezani et al., 2023):

$$\begin{array}{ll} L\left(x_{1},x_{2},\cdots ,x_{n},\lambda_{1},\cdots ,\lambda_{m}\right) & =f\left(x_{1},x_{2},\cdots ,x_{n}\right)+\sum_{i=1}^{m}\lambda_{i}r_{i}\\ \frac{\partial L}{\partial x_{i}} & =0,\;i=1,\cdots ,n\\ \frac{\partial L}{\partial \lambda_{j}} & =0,\;j=1,\cdots ,m \end{array}$$ (16)

A new and innovative RNN structure has been proposed to find a near-optimal solution in possible movement directions. This structure is made up of newly designed Gaussian spiking neurons that construct the cost function criteria concept in their membrane function. The synaptic neurons are defined in such a way that the network can search for the best position.

The main idea behind this proposed structure is to solve the Lagrangian problem by considering synaptic current as a term that can indicate the weighted constraints. This synaptic current is then inputted to the synaptic neurons.

**FIGURE 3:**
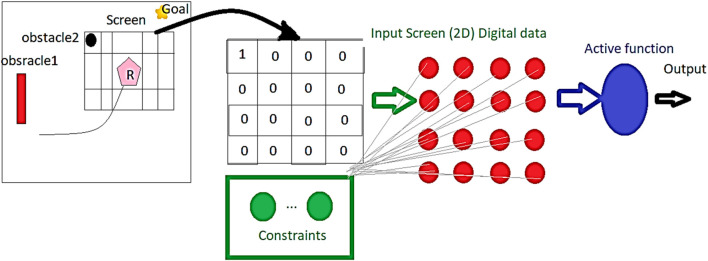
Proposed neural navigator structure.

Figure 3 demonstrates how the searching screen window is selected based on the location of the robot and the prediction horizon. Additionally, the figure indicates the constraints inputted to the reservoir recurrent neural network beside the selected window. The amount of four-sided shapes was based on the desired forecast range, with a single quadrangle corresponding to a forecast one step ahead, two quadrangles corresponding to a forecast two steps ahead, and so on. This facilitated the capture of pertinent data pertaining to the automaton’s immediate surroundings at various prediction intervals, which was employed as input to our RNN-based optimization methodology. The dimensions of the display were determined in accordance with the number of quadrangles required to capture the sought-after prediction horizon.

After analyzing the low-risk position corresponding to the minimum membrane potential, the activation function generates the desired path according to the calculated feedback gain corresponding to the low-risk possible location. In other words, the minimum Vn indices indicate the path error feedback indices, and consequently, the desired path is calculated.

The proposed network algorithm is defined in Eq. 17. This new RNN structure has the potential to revolutionize the way we approach movement directions and find optimal solutions.

$$\begin{array}{l} V_{n}=\sum_{i=x_{r}-1}^{x_{r}+1}\sum_{j=y_{r}-1}^{y_{r}+1}s_{ij}e^{-{\left(x_{r}-i-1\right)}^{2}-{\left(y_{r}-j-1\right)}^{2}}+\left(2s_{ij}-1\right)e^{-{\left(x_{f}-i\right)}^{2}-{\left(y_{f}-j\right)}^{2}}+I_{n}\\ \rho_{n}=\sum_{k=1}^{m}W_{nk}r_{k}+\theta_{n}\\ I_{n}=\tanh\left(\rho_{k}\right)\\ r_{1}=\Delta x_{r}-V_{x\max}.\Delta t\\ r_{2}=\Delta y_{r}-{V_{y}}_{\max}.\Delta t\\ r_{3}=x_{r}-\gamma \varphi \left[1\right]\\ r_{4}=y_{r}-\gamma \varphi \left[2\right]\\ r_{6}=\tan^{-1}\left(y/x\right)-Flag\times \frac{\pi }{4}\\ r_{7}=1-\left(-Y^{T}\overset{Z}{\overbrace{\Lambda^{-1}\left(X_{oi}-Y_{f}\right)}}+\left(Y^{T}\right)\overset{\Phi }{\overbrace{F^{T}\Lambda^{-1}F}}\left(Y-Y_{f}\right)\right) \end{array}$$ (17)

where *S*_*ij*_ is a Boolean value that represents the state of a grid cell in the global screen. The screen is divided into a grid based on the scale of the robot’s dimensions, treating the robot as a single point in the encoded matrix of the environment. The values of *x*_*r*_ and *y*_*r*_ represent the robot’s position in the (x-y) coordinate system. The variable *V*_*n*_ in the equation represents the cost of each state in the neighborhood of the robot, as well as the cost of searching around it, considering one-step movements and calculating the cost of the distance from the target. *S*_*ij*_ is used to increase or decrease the cost if an obstacle is present or absent at that position. The first term of *V*_*n*_ searches for low-risk positions around the robot, while the next term evaluates the risk of one-step movements.

This searching structure is designed with the aim of reducing map size dependency and consequently its computational cost. In is the input synaptic current on the neuron n and Nv is the neuron membrane function, which indicates the proposed cost function and 1 step prediction of moving environment screen. The combination of the searching-based idea with the reservoir structure can reduce sensitivity of the proposed navigator to uncertainties and improve its robustness.

The input neurons and the size of proposed network can be considered constant and relevant to the steps of prediction horizon. Synaptic weights (*W*_*ij*_) are updated based on minimizing *V*_*n*_, as an indicator of the *(i, j)*th Lagrangian function of the proposed algorithm.

$$\begin{array}{ll} ∆W_{nk} & =-\eta \frac{\partial V_{n}}{\partial W_{nk}}=-\eta \left(1-\rho_{n}^{2}\right)r_{k}\\ ∆\theta_{n} & =-\eta \left(1-\rho_{n}^{2}\right)\\ ∆F & =-\mu \frac{\partial r_{7}}{\partial \Phi }=\mu \Lambda^{\frac{1}{2}}{\left({YY}^{T}-{YY}_{f}^{T}\right)}^{\frac{1}{2}} \end{array}$$ (18)

The desired error feedback gain is calculated based on the minimum-Vn neuron location. Consequently, the desired path is calculated according to the following active function:

$$Y_{d}=\left\{\begin{array}{ll} Y_{robot}+F\left(Y_{f}-Y_{robot}\right) & \left|Y_{f}-Y_{d}\right|>2V_{\max}*dt\\ Y_{f} & else \end{array}\right.$$ (19)

## 3 Simulation results

In order to assess the effectiveness of our proposed navigation strategy, we conducted simulations using Python 3.10. We simulated a real three-wheeled omnidirectional mobile robot dynamic model, which included a built-in tracking controller as introduced by (Peñaloza-Mejía et al., 2015). These simulations were conducted in various dynamic and static environments. The field of robotics necessitates intricate path planning that encompasses a multitude of factors leading to indeterminate outcomes, including sensor noise, modeling inaccuracies, extraneous interferences, and environmental disturbances. These uncertainties can be typified based on their origin, nature, conduct, and scope. The study considers white noise with normal (0, 1) for the sensors’ noise, prediction error between 0 and 2 times greater than robot diameter, and random velocity in the obstacles’ movements.

We compared the performance of our newly designed log-concave strategy, which utilizes a recurrent neural network, with the results obtained from the CVXPY optimization library, as well as the RRT, LQR, and A-star navigation methods. Through these simulations, we aimed to answer the following questions and evaluate the performance of our proposed strategy:

- How does our proposed strategy compare to existing navigation methods?
- How does our strategy perform in dynamic and static environments?
- What are the strengths and weaknesses of our proposed strategy?

The comparison was made to provide a benchmark for the performance of the proposed algorithm in terms of computational efficiency and path quality. While the approaches are fundamentally different, both methods have the ability to search in the neighborhood for feasible paths. A*/RRT use a search algorithm to explore neighboring nodes to find a suitable path, while the proposed RNN structure uses a searching screen window to identify low-risk positions for path planning.

To evaluate the performance of each method, we conducted a series of simulations in dynamic and static environments. We compared the computational efficiency and path quality of the proposed RNN structure with that of A*, RRT, LQR, and the proposed log-concave MPC using CVXPY optimization library. The metrics used to evaluate performance included the computation time, path length, and collision avoidance ability.

### 3.1 Impact of dynamic constraints on path planning efficiency

The dynamics of a robot can limit its ability to follow a planned path precisely, as shown in Figure 3.

**FIGURE 4:**
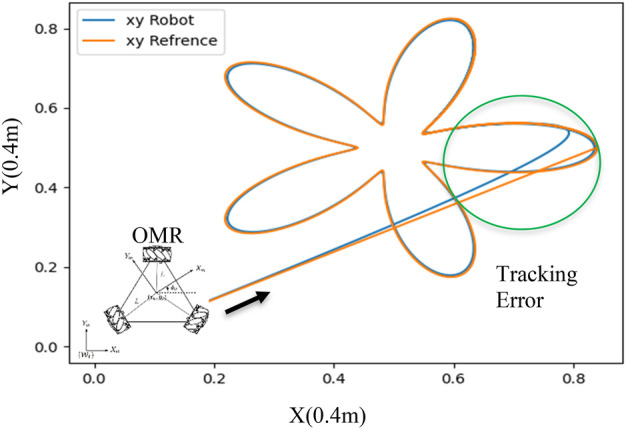
Path Tracking and Errors due to the robot’s dynamic and internal controller performance introduced by (Peñaloza-Mejía et al., 2015).

Figure 4 exemplifies the plausible path tracking predicaments that mobile robot may face, encompassing inaccuracies and aberrations. These predicaments may culminate in the fiasco of collision evasion schemes, especially in fluctuating surroundings where barriers approach the automaton. To ensure the safe and efficacious operation of mobile automatons, it is crucial to ponder over the impact of dynamic constraints on the efficiency of path plotting. By apprehending the restrictions imposed by a robot’s dynamics, we can formulate more sturdy and dependable path plotting algorithms that take into account the possibility of inaccuracies. This approach can decrease the probability of collisions and other safety perils while augmenting the comprehensive efficiency and performance of mobile robots.

### 3.2 Performance of the proposed log-concave MPC in sample environments with static and dynamic obstacles

To assess the effectiveness of the proposed navigation strategy, we utilized the CVXOPT library to solve the constrained MPC problem. Our evaluation included sample environments with both static and dynamic obstacles, all within a 20 × 20 screen size.

**FIGURE 5:**
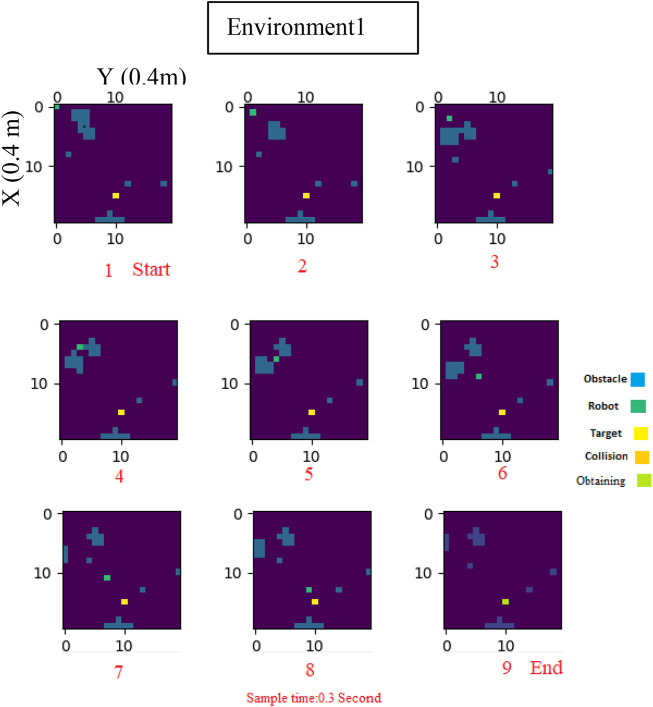
Log-Concave MPC path planning in dynamic Environment1.

Figure 5 provides a visual representation of the robot’s movements and the obstacles encountered every 0.3 s. As demonstrated in this figure, the robot successfully navigated to its target destination without any collisions.

**FIGURE 6:**
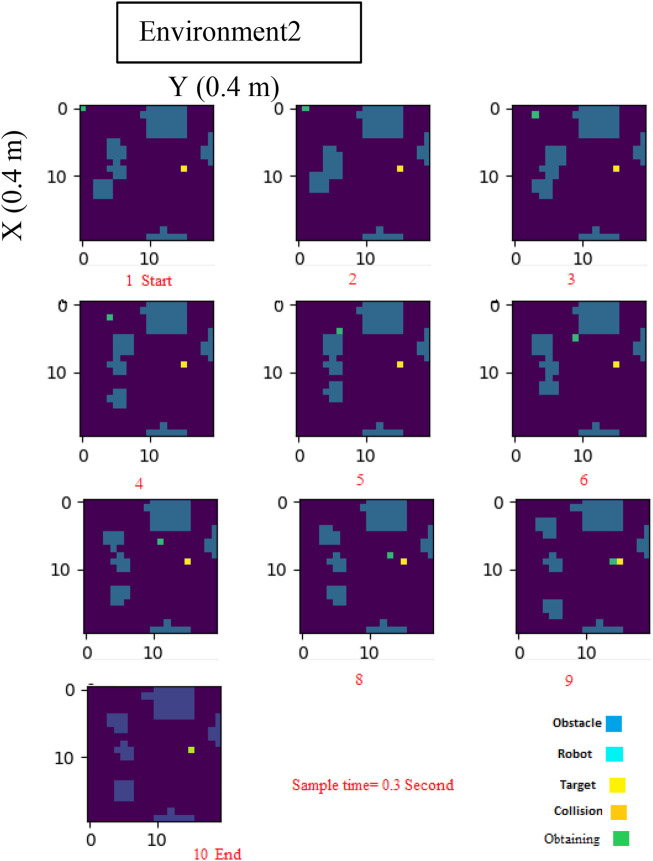
Log-Concave MPC path planning in dynamic Environment2.

To further evaluate the efficiency of our proposed strategy, we utilized Figure 6, which provides a global overview of the environment. Through these evaluations, we were able to demonstrate the effectiveness of our log-concave MPC approach in navigating mobile robots through environments with both static and dynamic obstacles.

### 3.3 Difference between the designed RNN planner and the optimization of log-MPC strategy

This section evaluates the performance of RNN searching window and its efficiency in comparison to utilize CVXOPT optimization tool in another example of environment.

CVXOPT is a convex optimization library that provides solvers for a wide range of convex optimization problems, including nonlinear MPC. The specific algorithm used by CVXOPT for solving the nonlinear MPC problem depends on the formulation of the problem and the specific options selected by the user. In general, CVXOPT uses a primal-dual interior-point method for solving convex optimization problems (Dahl and Andersen, 2022). This method iteratively solves a sequence of linear systems and updates the primal and dual variables until convergence. For nonlinear MPC problems, CVXOPT can use either a direct or an indirect method to solve the problem.

**FIGURE 7:**
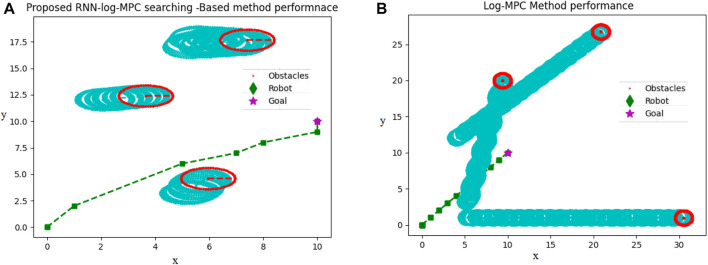
**(A)** Proposed RNN-Log-MPC navigation performance in a dynamic environment **(B)** Proposed Log-MPC navigation performance in a dynamic environment, utilizing CVXOPT optimization tool. Unit is 0.4 m, corresponding to the robot dimension.

Figure 7 reveals how both proposed path planning methods navigate the robot to destination, safely. This comparison has been conducted in the same dynamic environment, in which three dynamic environments start moving from the same initial points in both a and b figures and the obstacle’s velocity are also considered the same.

**FIGURE 8:**
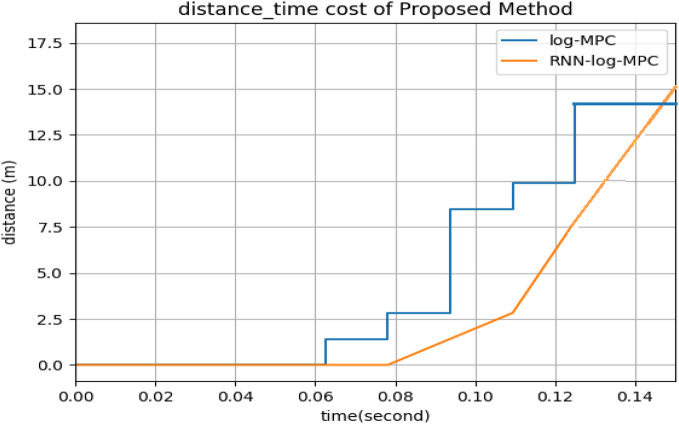
Comparison of Distance-time cost between Log-MPC and RNN-Log-MPC.

In Figure 7, the path passed by the obstacles is depicted by light blue circles. The current positions of obstacles are depicted in red circles. The robot passes through a green–square dash line, and the star is the target destination. Upon scrutinizing both Figures 7A, B, it becomes conspicuous that the simulation circumstances are strikingly alike. The motion of obstacles and their velocity remains consistent in both illustrations. However, a noticeable dissimilarity arises when contemplating the obstructions illustrated in Figure 7B, as they progressively shift further away from the intended target locale in comparison to Figure 7A. Therefore, the robot in Figure 7B is coerced to traverse for an extended duration in order to attain the desired position. This incongruity can be credited to the inferior pace of the robot in Figure 7B, which, in turn, is instigated by the path planner dispensing a smaller displacement by CVXOPT. In this situation, some generated paths of movements are in the range of 0.03 which cannot empower the robot efficiently, therefore, the robot stays at its position for several sequential sample times. In contrast, the initial training time of the RNN takes almost 0.05 (second) longer than the CVXOPT tool. Then, the robot is continuously navigated to the target position without any long time stopping in a particular position. Of course, we cannot regard stopping in a particular position as a disadvantage for a path planner, while in social environments, waiting for other people to pass the way and then continuing the optimal path or safe route is considered an advantage. On the other hand, this feature is not recommended in automated vehicles on highways, in air traffic, and other applications. In this case, RNN-Log-MPC can be more efficient. Figure 8 indicates the time-distance cost comparison.

### 3.4 Performances of the proposed log-MPC and RNN-log-MPC strategies in comparison of RRT, A-star and LQ-MPC methods

**FIGURE 9:**
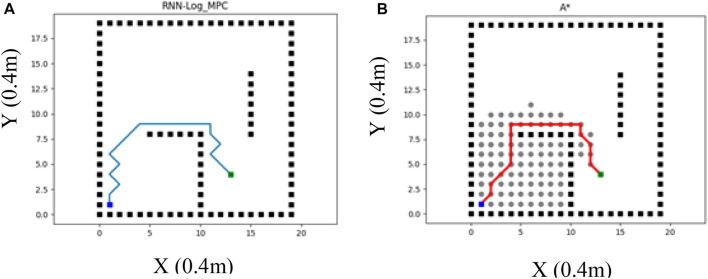
**(A)** RNN-Log-MPC path planning method in a static environment **(B)** A^*^ method performance in a static environment (the left figure).

Classic methods are not able to handle path planning programming in dynamic environments efficiently. Therefore, RRT and A-star as the classic methods are recommended to be utilized in static environments. On the other hand, reactive and heuristic methods are mostly utilized in dynamic environments. To evaluate the performance of the proposed Log-MPC method, the following Table 2 depicts the comparison of RNN-Log-MPC and A-star performances in the given below static environment (Figure 9).

**TABLE 2:** RRT, A-star, LQ-MPC, and the Proposed Method (RNN-Log-MPC) Comparison in, including 3 moving obstacles and the same target point.

Methods	Distance cost	Time cost	Collision with obstacle	Concern
RNN-Log-MPC	15	0.14	No	Dependency on accurate prediction
Log-MPC using CVXPY	15	0.16	No	Infeasibility and dependency on accurate prediction
RRT	24	0.2	Yes	No solution in some cases and failing in dynamic environment
A^*^	17	0.157	Yes	Collision and high computational cost in real time applications
LQ-MPC (Werling et al., 2010)	15.8	0.153	Yes	Cannot find solution in long distance target situations

Figure 9 illustrates a static environment example in which RNN-log-MPC method is compared with A^*^ strategy. The proposed strategy is considered with one prediction step as a global planner. The costs are compared in Table 2. Table 2 provides a comparison of both strategies in the same environment and with the same processor, significant efficiency of the proposed method, especially in the sense of time costs. In addition, the results illustrate that RRT may fail in providing a free-collision path in a dynamic environment, while the proposed method can navigate the robot efficiently.

The parameters of simulated RNN is given in the Table 3:

**TABLE 3:** RNN parameters.

Items	Quantity
$\rho_{n}$	0.1
η	0.7
μ	0.8

Another sample environment includes two sinusoidal shape movements of the obstacles and one random walker, with the target position at the point (12, 15). The robot movement path is simulated under the following conditions:

1.When the utilized path planning algorithm is RRT.
2.When the utilized path planning algorithm is the new proposed Navigator.

**FIGURE 10:**
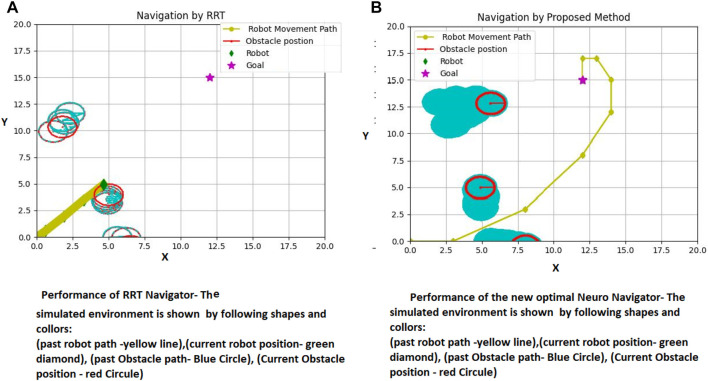
Proposed method and RRT Path Planning in a dynamic environment (fourth Example), **(A)** RRT Performance **(B)** Proposed Method Performance.

The global screen of the environment, including the movement path of the robot and obstacles are shown in Figure 10.

**FIGURE 11:**
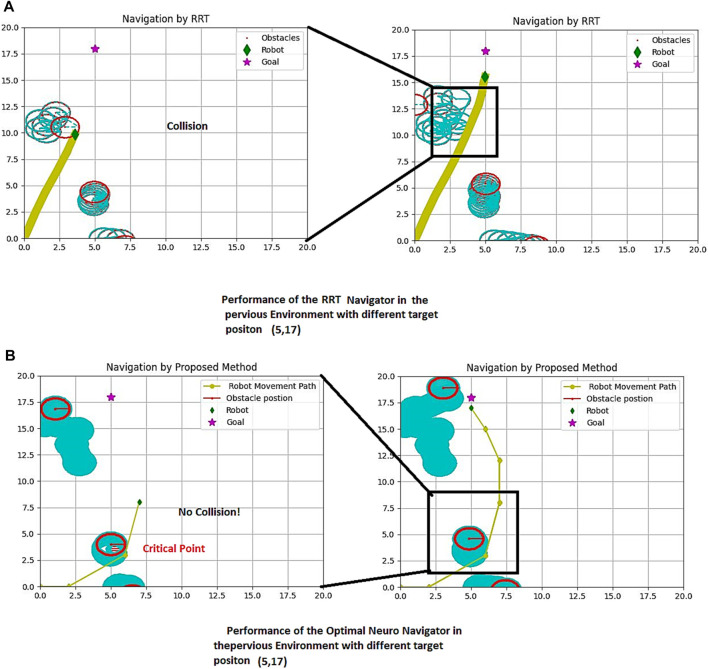
Proposed method and RRT Path Planning in the same previous dynamic environment with different target. **(A)** RRT performance **(B)** The proposed Method Performance.

Figure 11 presents a more comprehensive comparison of our proposed method and RRT in a simulated environment with varying target positions. The figure showcases the success of our method and the failure of RRT. The target is represented by a violet star shape, while the robot is depicted as a green diamond. The obstacles are shown as red circles, and the passed path is indicated by the yellow line. The left side of both parts of Figure 10A demonstrates the crucial scenes, while the right side shows the entire passed trajectory.

It is evident that generating a path using RRT can be time-consuming, especially if a unique solution is obtainable in this structure. To better understand this issue, we have provided a table below that compares our proposed method with RRT in terms of distance versus time. The simulation screen size is 20*20 with a scale of 0.45 m, and the arrangement of obstacles differs in at least five similar simulation examples. The target position is located at (Limon et al., 2017; Kim et al., 2018) in Figures 10, 11.

Our proposed method offers a more efficient and effective solution for navigation in a simulated environment. With our method, the robot can navigate through the obstacles and reach the target in a shorter amount of time compared to RRT. This not only saves time but also reduces the risk of errors and increases the overall success rate of the navigation process.

Figures 10A, B demonstrates a comparison of the RRT performance (10-a) with the suggested strategy (10-b). The results show that the RRT failed in providing a safe path, and consequently collides with the obstacle in a dynamic environment.

Figure 11 presents a more comprehensive comparison of our proposed method and RRT in a simulated environment with varying target positions. The figure showcases the success of our method and the failure of RRT. The target is represented by a violet star shape, while the robot is depicted as a green diamond. The obstacles are shown as red circles, and the passed path is indicated by the yellow line. The left side of both parts of Figure 10A demonstrates the crucial scenes, while the right side shows the entire passed trajectory.

It is evident that generating a path using RRT can be time-consuming, especially if a unique solution is obtainable in this structure. To better understand this issue, we have compared our proposed method with RRT in terms of distance versus time in Table 2. The simulation screen size is 20*20 with a scale of 0.45 m, and the arrangement of obstacles differs in at least five similar simulation examples. The target position is located at (Limon et al., 2017; Kim et al., 2018) in Figures 10, 11.

Our proposed method offers a more efficient and effective solution for navigation in a simulated environment. With our method, the robot can navigate through the obstacles and reach the target in a shorter amount of time compared to RRT. This not only saves time but also reduces the risk of errors and increases the overall success rate of the navigation process.

### 3.5 Potential challenges and future directions

The proposed RNN-Log-MPC algorithm presents a notable advantage by utilizing both sensor and camera data to diminish the sensitivity of the navigation system to momentary data loss or prediction error by EKF. Nonetheless, this does not eliminate the exigency to tackle potential issues such as prediction errors, sensor disconnection, and lack of accuracy in the command follower controller. Therefore, a conservative approach to adjusting and evaluating restrictions may be deemed necessary, which could constrain optimization, particularly in unfamiliar environments where speed changes occur, and there is a possibility of collision risk.

Moreover, the precision of obstacle detection, which is processed via a central server and communicated wirelessly to the robot’s internal controller, can also influence the system’s performance. Hence, it is crucial to scrutinize the algorithm in various scenarios to identify potential errors and devise solutions to rectify them. Constant monitoring of the system can also enhance the accuracy of performance and reliability of the navigation system.

## 4 Conclusion

This study proposes a groundbreaking navigation algorithm that employs a log-concave strategy and a visual-based model predictive controller (MPC) framework. The tactic is innately near-optimal and restricted, and it is tackled by employing two optimization resources, namely, a newly proposed network configuration and the CVXOPT optimization library. The suggested recurrent neural network (RNN) architecture adeptly resolves diverse constrained navigation predicaments. The constraints, which include the velocities of the robot and the nearby obstacles, are given careful consideration to enhance the reliability of the navigation system. Our simulations demonstrate promising results, but further research is imperative to scrutinize the applicability of our methodology in pragmatic settings or using tangible mechanisms such as Gazebo or VREP to validate our approach and provide additional insights.

This investigation furnishes a resolution to the optimal visual navigation technique, where the structure size remains constant in various environments with different numbers of obstacles, by utilizing a window-based searching approach. Nonetheless, the Extended Kalman Filter, which is utilized to localize objects, is a highly favored stochastic method in this area, but it operates based on the linearization method. Consequently, there is still a concern about its failing events, which can trigger low-accurate predictions of the environment.

To summarize, our proposed RNN-based optimization methodology for circumventing obstacles showcases auspicious outcomes and harbours the potentiality of being expanded to more intricate scenarios and environments. For instance, our methodology may be implemented in pragmatic situations such as self-governing motoring, wherein the robot necessitates traversing through a multifarious and dynamic milieu while evading hindrances. Furthermore, our approach has the capacity to amalgamate with alternative state estimation and prognostication techniques to advance the performance and resilience of the system. We are of the conviction that our study lays a robust groundwork for ensuing research in the establishment of RNN-based techniques for circumventing obstacles in autonomous robots.

## Figures and Tables

**Table:** 

Nomenclature entries should have the units identified	
***M***	Mass
***r***	Wheels’ radius
***L***	Distance to wheels
***I***_***z***_	Inertia of the Robot
***J***	Inertia of the Wheels
***d***	Maximum possible movement
$d_{\theta }$	Maximum possible rotation angle
***V***_***max***_	Maximum possible velocity for the robot
$\omega_{\max}$	Maximum possible angular velocity
${\hat{\gamma }}^{T}$	The estimated model parameter by Kalman Filter
${\hat{\varphi }}_{w}\left(k\right)$	The estimated robot angle
${\hat{Y}}_{k+1}$	The one-step-ahead prediction of $Y_{k}$
$Y_{f}$	The (x-y) position vector of the target destination
$Y_{d}\left(k\right)$	The generated path at time step *k*
$S_{ij}$	The Boolean number 0 or 1 of the screen state in *(i,j)*_th_ matrix index
$I_{n}$	The input synaptic current on the neuron n (mA)
$\rho_{n}$	The adaption potential (mV)
***V***_***n***_	The neuron membrane function (mV)
Subscripts	
$d_{\theta }q={\left[\begin{array}{ccc} x_{w} & y_{w} & \varphi_{w} \end{array}\right]}^{T}$	Position of the robot in the global coordinate and angle of the robot vector
${}^{,}q$	Robot velocity
${}^{∙}q$	Robot acceleration
$\tau \in R^{3}$	Torque input
$M\left(q\right)\in R^{3}$	Inertial matrix
$C\left(q^{∙},q^{∙}\right)q$	Coriolis
$B\left(q\right)\in R^{3\times 3}$	Centrifugal force vector
$x_{oi\left(t-1\right)}$	The *i*th obstacle position
$v_{oi\left(t-1\right)}$	The *i*th obstacle velocity
$J\left(t\right)$	The cost of the MPC at time t
$Y_{k}=Y\left(t\right)={\left[q^{∙}q\right]}^{T}$	Sample of position and velocity vector of the robot dynamic at time t = k∆t
***U***_***k***_	The control law, i.e., the generated path
${\hat{Y}}_{k+1}$	The one-step-ahead prediction of $Y_{k}$
***F***	Discrete-time error-feedback gain

## Data Availability

The simulation data used in this study are not publicly available due to confidentiality reasons. However, the authors can provide access to the data upon request and after appropriate agreements are made. Please contact the corresponding author for more information or inquiries regarding the data.

## References

[B1] Achirei S.-D. Mocanu R. Popovici A.-T. Dosoftei C.-C. (2023). Model-predictive control for omnidirectional mobile robots in logistic environments based on object detection using CNNs. Sensors 23 (11), 4992. 10.3390/s23114992 37299719 PMC10255710

[B2] Askari I. Badnava B. Woodruff T. Zeng S. Fang H. (2022). “Sampling-based nonlinear MPC of neural network dynamics with application to autonomous vehicle motion planning,” in Proceedings of the 2022 American Control Conference (ACC), Atlanta, GA, USA, June 2022, 2084–2090.

[B3] Bency M. J. Qureshi A. H. Yip M. C. (2019). “Neural path planning: Fixed time, near-optimal path generation via oracle imitation,” in Proceedings of the 2019 IEEE/RSJ International Conference on Intelligent Robots and Systems (IROS), Macau, China, November 2019, 3965–3972.

[B4] Blanchard E. (2007). “Parameter estimation method using an extended Kalman filter,” in Proceedings of the Joint North America, Asia-Pacific ISTVS Conference, Fairbanks, Alaska, USA, June 2007.

[B5] Cheng S. Li L. Guo H.-Q. Chen Z.-G. Song P. (2019). Longitudinal collision avoidance and lateral stability adaptive control system based on MPC of autonomous vehicles. IEEE Trans. Intelligent Transp. Syst. 21 (6), 2376–2385. 10.1109/tits.2019.2918176

[B6] Dahl J. Andersen E. D. (2022). A primal-dual interior-point algorithm for nonsymmetric exponential-cone optimization. Math. Program. 194 (1-2), 341–370. 10.1007/s10107-021-01631-4

[B7] Deits R. Tedrake R. (2014). “Footstep planning on uneven terrain with mixed-integer convex optimization,” in Proceedings of the 2014 IEEE-RAS international conference on humanoid robots, Madrid, Spain, November 2014, 279–328.

[B8] Du H. Hao B. Zhao J. Zhang J. Wang Q. Yuan Q. (2022). A path planning approach for mobile robots using short and safe Q-learning. Plos one 17 (9), e0275100. 10.1371/journal.pone.0275100 36162062 PMC9512417

[B9] Egmont-Petersen M. de Ridder D. Handels H. (2002). Image processing with neural networks—A review. Pattern Recognit. 35 (10), 2279–2301. 10.1016/s0031-3203(01)00178-9

[B10] Haider M. H. Wang Z. Khan A. A. Ali H. Zheng H. Usman S. (2022). Robust mobile robot navigation in cluttered environments based on hybrid adaptive neuro-fuzzy inference and sensor fusion. J. King Saud University-Computer Inf. Sci. 34 (10), 9060–9070. 10.1016/j.jksuci.2022.08.031

[B11] Karur K. Sharma N. Dharmatti C. Siegel J. E. (2021). A survey of path planning algorithms for mobile robots. Vehicles 3 (3), 448–468. 10.3390/vehicles3030027

[B12] Kim Y.-H. Jang J.-I. Yun S. (2018). “End-to-end deep learning for autonomous navigation of mobile robot,” in Proceedings of the 2018 IEEE International Conference on Consumer Electronics (ICCE), Las Vegas, NV, USA, January 2018, 1–6.

[B13] Lee M.-F. R. Yusuf S. H. (2022). Mobile robot navigation using deep reinforcement learning. Processes 10 (12), 2748. 10.3390/pr10122748

[B14] Limon D. Calliess J. Maciejowski J. M. (2017). Learning-based Nonlinear Model Predictive Control * *The authors would like to ackowledge to the Spanish MINECO Grant PRX15-00300 and projects DPI2013-48243-C2-2-R and DPI2016-76493-C3-1-R as well as to the Engineering and Physical Research Council, grant no. EP/J012300/1 for funding this work. IFAC-PapersOnLine 50 (1), 7769–7776. 10.1016/j.ifacol.2017.08.1050

[B15] Peñaloza-Mejía O. Márquez-Martínez L. A. Alvarez J. Villarreal-Cervantes M. G. García-Hernández R. (2015). Motion control design for an omnidirectional mobile robot subject to velocity constraints. Math. Problems Eng. 2015, 1–15. 10.1155/2015/608015

[B16] Quan H. Li Y. Zhang Y. (2020). A novel mobile robot navigation method based on deep reinforcement learning. Int. J. Adv. Robotic Syst. 17 (3), 172988142092167. 10.1177/1729881420921672

[B17] Ramezani M. Habibi H. Sanchez-Lopez J. L. Voos H. (2023). “UAV path planning employing MPC-reinforcement learning method considering collision avoidance,” in Proceedings of the 2023 International Conference on Unmanned Aircraft Systems (ICUAS), Warsaw, Poland, June 2023, 507–514.

[B18] Saeedinia S. A. Tale Masouleh M. (2022). The synergy of the multi-modal MPC and Q-learning approach for the navigation of a three-wheeled omnidirectional robot based on the dynamic model with obstacle collision avoidance purposes. Proc. Institution Mech. Eng. Part C J. Mech. Eng. Sci. 236 (17), 9716–9729. 10.1177/09544062221095414

[B19] Salzmann T. Kaufmann E. Arrizabalaga J. Pavone M. Scaramuzza D. Ryll M. (2023). Real-time neural MPC: Deep learning model predictive control for quadrotors and agile robotic platforms. IEEE Robotics Automation Lett. 8 (4), 2397–2404. 10.1109/lra.2023.3246839

[B20] Song Y. Scaramuzza D. (2020). “Learning high-level policies for model predictive control,” in Proceedings of the 2020 IEEE/RSJ International Conference on Intelligent Robots and Systems (IROS), Las Vegas, NV, USA, October 2020, 7629–7636.

[B21] Stano P. (2022). Model predictive path tracking control for automated road vehicles: A review. Annu. Rev. Control.

[B22] Villarrubia G. De Paz J. F. Chamoso P. De la Prieta F. (2018). Artificial neural networks used in optimization problems. Neurocomputing 272, 10–16. 10.1016/j.neucom.2017.04.075

[B23] Wang J. Ding X. Xia H. Wang Y. Tang L. Xiong R. (2017). “A LiDAR based end to end controller for robot navigation using deep neural network,” in Proceedings of the 2017 IEEE International Conference on Unmanned Systems (ICUS), Beijing, China, October 2017, 614–619.

[B24] Werling M. Ziegler J. Kammel S. Thrun S. (2010). “Optimal trajectory generation for dynamic street scenarios in a frenet frame,” in Proceedings of the 2010 IEEE International Conference on Robotics and Automation, Anchorage, AK, USA, May 2010, 987–993.

[B25] Xie R. Meng Z. Wang L. Li H. Wang K. Wu Z. (2021). Unmanned aerial vehicle path planning algorithm based on deep reinforcement learning in large-scale and dynamic environments. IEEE Access 9, 24884–24900. 10.1109/access.2021.3057485

[B26] Xiao X. (2022). “Learning model predictive controllers with real-time attention for real-world navigation,", 2022, https :// arxiv.org /abs/2209.10780.

[B27] Yang K. Gan S. K. Sukkarieh S. (2010). An efficient path planning and control algorithm for RUAV’s in unknown and cluttered environments. J. Intelligent Robotic Syst. 57, 101–122. 10.1007/s10846-009-9359-1

[B28] Yao P. Wang H. Su Z. (2015). Real-time path planning of unmanned aerial vehicle for target tracking and obstacle avoidance in complex dynamic environment. Aerosp. Sci. Technol. 47, 269–279. 10.1016/j.ast.2015.09.037

